# Highly Graphitized Carbon Coating on SiO with a π–π Stacking Precursor Polymer for High Performance Lithium-Ion Batteries

**DOI:** 10.3390/polym10060610

**Published:** 2018-06-04

**Authors:** Shan Fang, Ning Li, Tianyue Zheng, Yanbao Fu, Xiangyun Song, Ting Zhang, Shaopeng Li, Bin Wang, Xiaogang Zhang, Gao Liu

**Affiliations:** 1 Energy Storage and Distributed Resources Division, Energy Technologies Area, Lawrence Berkeley National Laboratory, Berkeley, CA 94720, USA; fangshan_joy@163.com (S.F.); ningli@lbl.gov (N.L.); tyzheng@lbl.gov (T.Z.); yanbaofu@lbl.gov (Y.F.); x_song@lbl.gov (X.S.); zhangting@bit.edu.cn (T.Z.); wangbin0502@163.com (B.W.); 2 Jiangsu Key Laboratory of Electrochemical Energy Storage Technologies, College of Material Science and Engineering, Nanjing University of Aeronautics and Astronautics, Nanjing 210016, China; 15150675098@163.com

**Keywords:** graphite carbon, silicon monoxide, anode, coating, lithium-ion battery

## Abstract

A highly graphitized carbon on a silicon monoxide (SiO) surface coating at low temperature, based on polymer precursor π–π stacking, was developed. A novel conductive and electrochemically stable carbon coating was rationally designed to modify the SiO anode materials by controlling the sintering of a conductive polymer, a pyrene-based homopolymer poly (1-pyrenemethyl methacrylate; PPy), which achieved high graphitization of the carbon layers at a low temperature and avoided silicon carbide formation and possible SiO material transformation. When evaluated as the anode of a lithium-ion battery (LIB), the carbon-coated SiO composite delivered a high discharge capacity of 2058.6 mAh/g at 0.05 C of the first formation cycle with an initial Coulombic efficiency (ICE) of 62.2%. After 50 cycles at 0.1 C, this electrode capacity was 1090.2 mAh/g (~82% capacity retention, relative to the capacity of the second cycle at 0.1 °C rate), and a specific capacity of 514.7 mAh/g was attained at 0.3 C after 500 cycles. Furthermore, the coin-type full cell composed of the carbon coated SiO composite anode and the Li[Ni_0.5_Co_0.2_Mn_0.3_O_2_] cathode attained excellent cycling performance. The results show the potential applications for using a π–π stacking polymer precursor to generate a highly graphitize coating for next-generation high-energy-density LIBs.

## 1. Introduction

Lithium-ion batteries (LIBs), consisting of graphite and lithium cobalt oxide (LiCoO_2_) electrodes, have been a major success in the consumer electronics industry due to their good stability and high performance. Higher energy density and long-term cycling stable and rechargeable LIB are needed in large-scale electrochemical energy storage systems, especially for electric vehicles and advanced power grids [1,2]. As a key component of LIBs, negative materials with improved storage capacity and thermal stability have been proposed to replacing graphite that has a theoretical capacity of only 372 mAh/g. Silicon and silicon-based anode material have been attracting the most research attention due to their unparalleled theoretical capacity (3579 mAh/g for Si and ~1500 mAh/g for SiO), relatively low discharge potential (<0.5 V vs. Li/Li^+^), abundant reserves, and low cost [3,4]. However, commercial application has been impeded by drawbacks in terms of the large volume changes that occur during lithiation and de-lithiation, thus disrupting the electrode integrity and breaking up the solid electrolyte interface (SEI). The breakdown of the SEI layer during cycling is one of the main reasons for large capacity fading, low initial Coulombic efficiency (ICE) during cycling, and poor cycling stability [5,6,7]. 

To address these challenges, engineered nano-structuring has been reported and proven to be successful in promoting electrochemical performance. Various delicate nanostructures have been designed and fabricated, such as yolk-shell [8,9,10], pomegranate-like [11,12], nanotubes [13,14,15], and hollow spheres structure [16,17,18]. Although this void-in nanostructure can effectively accommodate the large volume changes and extend the cycle life, other new fundamental challenges related to the nanostructured electrodes have been introduced, including higher surface area, low tap density, complex synthesis process, and generally poor electrical properties due to the higher inter-particle resistance. 

The conductive polymer and carbon coating has been demonstrated to be a feasible approach to improve the electrochemical performance of the electrode materials for lithium-ion batteries. Conductive coating layers have been reported to not only increase the electrical conductivity, but also minimize the side reactions and minimize the volume changes as an electrolyte blocking layer on the surface of the Si-based material during cycling. For example, Yu et al. successfully synthesized a stable silicon anode material via the in-situ polymerization of polyaniline (PANi) to conformal coat silicon nanoparticles, and about 550 mAh/g was obtained after 5000 cycles at 6 A/g with a mass loading of 0.2–0.3 mg/cm [19]. Lee et al. used polyacrylonitrile (PAN) as a precursor, by limiting the pyrolysis temperature to 300–500 °C to attained the cyclization of PAN, which was achieved without carbonization while maintaining PAN’s polymeric properties. This uniform coating layer on the surface of Si nanoparticles has superior performance–nearly 1500 mAh/g has been achieved after 150 cycles at a current rate of 0.1 C [20]. Park et al. reported a nitrogen-doped carbon coating for SiO that exhibited substantially improved specific capacity and rate performance [21]. However, long-term cycling stability and high mass loading are still needed for these silicon-based anodes for practical applications. 

Here, we report using inexpensive and commercially available microparticles of silicon monoxide as a more promising electrode material for practical industrial applications. To create high stability and long-term cycling life of this silicon-based material, we describe an efficient yet easy strategy to modify the surface microstructure and electrical conductivity of SiO by introducing a high graphitization carbon agent to encapsulate the SiO at a mild temperature. The precursor, poly (1-pyrenemethyl methacrylate) (PPy) conductive polymer, was recently developed by our group as a functional conductive polymer adhesive binder [22]. The pyrene side chains of the PPy polymer form a π–π stacked structure in the solid-state to promote graphitization when the polymer is carbonized. Our experimental results demonstrate that the optimal pyrolysis of this polymer leads to high graphitization carbon coatings without formation of silicon carbide. Several reasons can support this unique coating on the surface of the SiO as an anode electrode: (1) the homogeneous encapsulation of the SiO by this graphite carbon can prevent direct contact between the electrolytes and the active material to restrain the repeatable formation of the SEI membrane and unfavorable parasitic reactions; (2) the graphite carbon layer can easily accommodate the strain and severe volume changes of the SiO generated during the cycling; and (3) the good electronic conductivity of the coating layer can facilitate rapid electronic transport on the surface of the SiO. The electrochemical tests indicated that the SiO encapsulated with this high graphitization coating layer has excellent cycling stability with high capacity and high coulombic efficiency (CE). Additionally, the full cells assembled with Li[Ni_0.5_Co_0.2_Mn_0.3_O_2_] (NCM) also demonstrated an excellent cycling performance. We hope this novel surface modification method will inspire the development of high-performance Si-based anode materials. 

## 2. Results and Discussion

**Figure 1:**
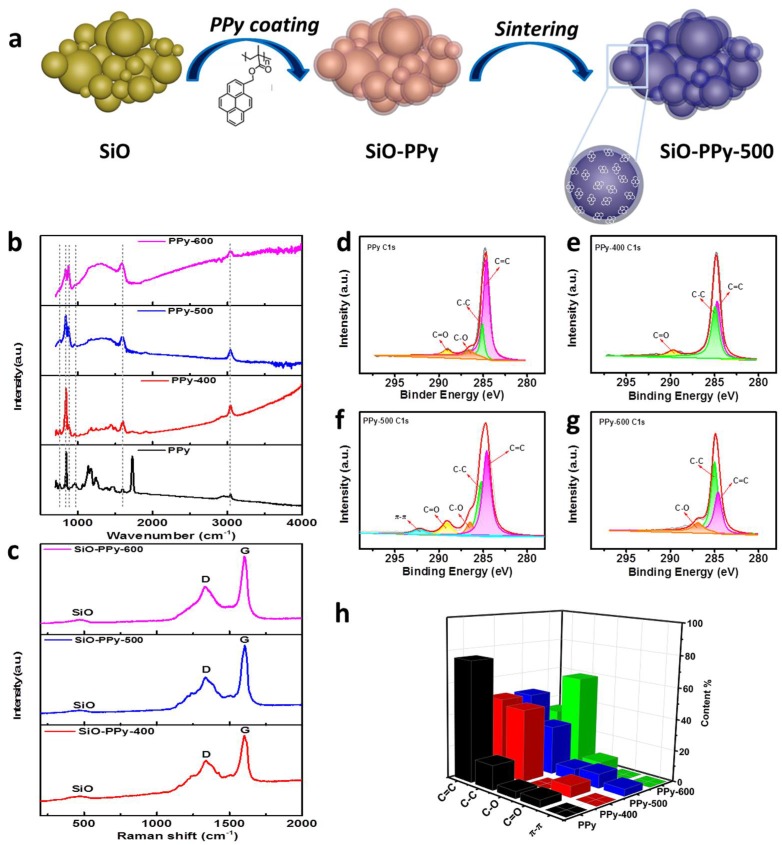
(**a**) Schematic illustrating the synthesis process of the SiO-PPy composite material sintered at different temperature; (**b**) Attenuated Total Reflectance Fourier transform infrared (ATR-FTIR) spectra of untreated PPy and PPy pyrolyzed at 400–600 °C; (**c**) Raman spectra of SiO-PPy treated at 400, 500, and 600 °C; (**d**–**g**) x-ray photoelectron spectroscopy (XPS) spectra; and (**h**) the corresponding carbon bonding composition of untreated PPy and PPy pyrolyzed at 400–600 °C.

A schematic illustration for the synthesis of the SiO-PPy composite material is shown in Figure 1a. We synthesized SiO–PPy sub-micrometer particles in a controlled manner. Typically, SiO sub-micrometer particles were dispersed in the solution of PPy in tetrahydrofuran (THF) and the resulting mixture was submitted to ultrasonics and stirred (the mass ratio of SiO and PPy is 7:3). Then, the solvents were evaporated by the rotary evaporator to obtain the SiO-PPy precursors, followed by sintering at low temperatures from 400 to 600 °C in an inert atmosphere to obtain the final products, which were uniformly coated with a carbon layer on the SiO surface. The coating process is simple, well-controlled, and consistent.

Attenuated Total Reflectance Fourier transform infrared (ATR-FTIR) and Raman were first used to characterize the structure transformation of the PPy during pyrolysis. Figure 1b shows the ATR-FTIR spectra of pristine PPy and PPy pyrolyzed between 400 and 600 °C. PPy before pyrolysis displayed bands at 1721 cm^−1^ due to C=O stretching of the ester groups, 1594 cm^−1^ due to the benzene skeleton vibration, and at 844 cm^−1^ due to aromatic stretching substitute. The bans at 1121 cm^−1^ is assigned to C–O–C stretching. When PPy was pyrolyzed between 400 and 600 °C, the peaks located at 1727 and 1121 cm^−1^ disappeared, which means the C–O–C bonds were cleaved [23,24]. The intensity of the peaks located at 1594 and 844 cm^−1^ decreased with the increase in pyrolysis temperature but did not totally disappear. The peaks at 3034 cm^−1^ (C-H stretching of benzene) is still visible for PPy-400 and PPy-500, meaning the pyrene is preserved after pyrolysis. In addition, the peak at 875 cm^−1^ corresponds to the substitution of benzene in PPy, even after pyrolysis at 400 and 500 °C. The other peak located at 755 cm^−1^ is attributed to the vibration of the methylene of the main chain. These results demonstrate that adjusting the pyrolysis procedure temperature for PPy can control the decomposition of the polymer and prevent fully carbonization of the polymer precursors. In the Raman spectra of the SiO-PPy-T composites (Figure 1c), two prominent peaks at 1340 and 1605 cm^−1^ were observed that correspond to the D and G band, respectively. Normally, the intensity ratio of the D and G bands (*I*_D_/*I*_G_) is used to determine defective disorders in the crystalline graphite or quality of graphitization. A lower I_D_/I_G_ ratio indicates better graphitization. For SiO-PPy-400, 500, and 600, the *I*_D_/*I*_G_ ratios were 0.65, 0.55, and 0.60, respectively, indicating that sintering at 500 °C resulted in the highest degree of graphite. To further understand the structure changes of PPy during pyrolysis, X-ray photoelectron spectroscopy (XPS) was used for analysis (Figure 1d–h). As shown in the C 1s spectra, the peaks corresponding to C–O, C–C, C=C, and C=O were found on the surface of the PPy and the pyrolyzed products, which agree with the ATR-FTIR results. The C=C content had a decreasing trend with the increase in temperature, which was almost the same at both 400 and 500 °C. The major difference lies in the spectra of C 1s at 500 °C: an extra peak was observed around 292.1 eV that was attributed to the delocalized *sp*2 π bonding, which enables good electronic conductivity [25,26]. This is also consistent with the Raman results: a stronger relative G band of the sample treated at 500 °C indicates a higher degree of ordered structure than the other samples. Therefore, we chose to focus the remainder of our material characterization on samples pyrolyzed at 500 °C.

**Figure 2:**
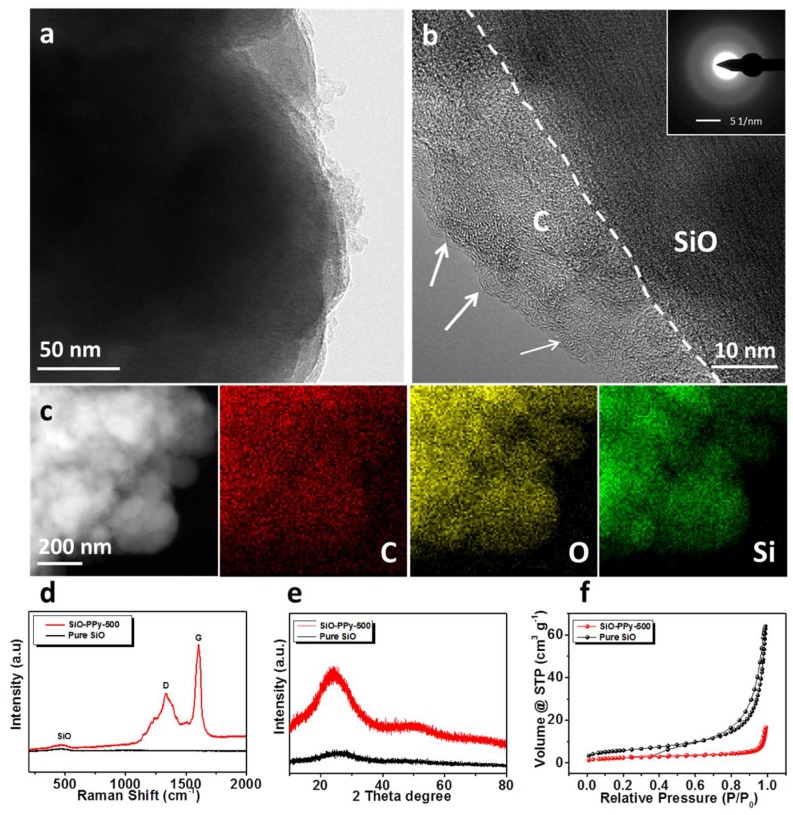
(**a**,**b**) High-resolution transmission electron microscopy (HRTEM) images of SiO–PPy–500. The top insert is the Selected Area Electron Diffraction (SAED) of the carbon; (**c**) Energy-dispersive X-ray spectroscopy (EDX) mapping of SiO-PPy-500; (**d**) Raman spectra of pure SiO and SiO–PPy–500; (**e**) x-ray diffraction (XRD) spectra of pure SiO and SiO–PPy–500 samples; and (**f**) the adsorption-desorption isotherm of pure SiO and SiO–PPy–500 composite.

The morphology of the composite samples was characterized by scanning electron microscopy (SEM) as shown in Figure S1. Compared with the pure SiO, SiO–PPy–500 has a similar structure before and after coatings. The particle size ranged from a few hundred nanometers to about one micrometer. When increasing the heating temperature to 500 °C, the outer surface of the particles became smoother after coating. The SiO–PPy–500 microstructure was further characterized by high-resolution transmission electron microscopy (HRTEM). The SiO particles were successfully coated with a layer of carbon with a thickness around 15 nm (Figure 2b). Notably, an ordered structure was observed on the edge of the particles. Based on previous research on the PPy polymer by our group, the ordered domains result from the π–π stacking of the pyrene units of the polymer. Additionally, an ordered precursor more easily creates a high graphite structure during sintering. Additional support for this uniform coating of the carbon layer is demonstrated by the dark-field scanning transmission electron microscopy (STEM) image and Energy-dispersive X-ray spectroscopy (EDX) mappings (Figure 2c). The results confirm that SiO was indeed wrapped by carbon. The elemental mapping images of silicon, carbon, and oxygen clearly demonstrate the uniform distribution of SiO in a carbon matrix. The Raman spectra of SiO–PPy–500 and pure SiO are shown in Figure 2d. A broad peak extending from 424 to 508 cm^−1^, with a peak at 465 cm^−1^, was observed, corresponding to the amorphous SiO. Additionally, two prominent peaks were observed at 1340 and 1605 cm^−1^, which correspond to the D and G bands, respectively. Figure 2e shows the XRD patterns of pure SiO particles and the composite samples of SiO-PPy-500. No diffraction peaks for pure SiO and SiO–PPy–500 were observed, confirming the amorphous structure of the samples, and the lack of silicon carbide formation for the SiO–PPy–500 composite. 

The surface areas of the SiO–PPy–500 composite and pure SiO were studied by N_2_ adsorption/desorption measurement (Figure 2f). According to the results, pure SiO has a surface area of 21.1 m^2^/g. After coating with PPy and pyrolysis, the surface area decreased to 8.6 m^2^/g for SiO-PPy-500, which can be attributed to extensive pore plugging due to the PPy pyrolysis. The low surface area helps to decrease the side reactions of SiO. Thermogravimetric analysis (TGA) was performed to identify the content of SiO in the composite samples. The TGA curve of SiO–PPy–500 in air is shown in Figure S2. The residual mass of SiO-PPy-500 at 700 °C is about 88.35 wt %. In addition, a slight weight increased occurred after 700 °C, indicating the oxidation of SiO under high temperatures. 

To evaluate the electrochemical performance of the SiO-PPy-500 composite, 2325-type coin cells were fabricated and the areal mass loading of SiO composite was controlled to be approximately 1.6–1.9 mg/cm^2^. Cyclic voltammetry (CV) was conducted between 2.0 and 0.01 V with a scan rate of 0.1 mV/s, as shown in Figure S3. A very weak cathodic peak appeared at 1.0–0.5 V (vs. Li/Li+) in the first onset, which is attributed to the SEI formation due to the reaction of the electrolyte with the surface of the active materials. The main cathodic peak in the first cycle, between 0.25 and 0.01 V, is attributed to the alloying of crystalline Si to form amorphous Li_x_Si phase. From the second cycle onward, a new cathodic peak appeared at ~0.1 V, indicating the reversible lithiation of the a-Si domains. Two anodic peaks were clearly observed at about 0.54 and 0.38 V. These peaks are associated with the de-alloying of the Li_x_Si. As the scan proceeded, the intensity of the anodic peaks increased, indicating the occurrence of the activation process of the sample. 

**Figure 3:**
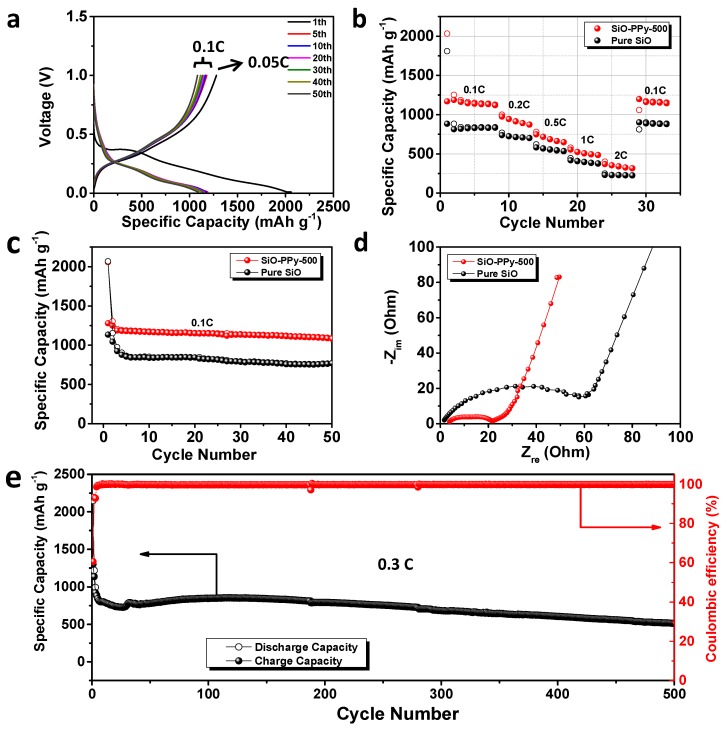
Electrochemical performance of the pure SiO and SiO–PPy–500 composite electrode: (**a**) discharge/charge profiles of SiO–PPy–500 measured at 0.05 C for the first cycle and then 0.1 C for 50 cycles; (**b**) the rate capabilities of pure SiO and SiO–PPy–500 at various current densities; (**c**) cycling performance at current densities of 0.1 C; (**d**) Nyquist plots of pure SiO and SiO–PPy–500 composite electrode after 50 cycles; and (**e**) long-term cycling performance of SiO-PPy-500 at the current rate of 0.3 C for 500 cycles.

The electrochemical properties of SiO-PPy-500 composite and pure SiO were systematically compared, and all the reported capacities in the half cell were calculated based on the mass of SiO. Its galvanostatic discharge-charge curves were obtained with a voltage window between 0.01 and 1.0 V versus Li/Li^+^ (Figure 3a) at a current rate of 0.05 C for the first cycle (1 C = 1000 mA/g current density). During the first SiO-PPy-500 discharge process, a broad and long slope rises with the voltage, ranging from 0.9 to 0.01 V in the first discharge, consistent with the electrochemical behavior of the typical Si-based composite electrode. The flat plateau at ~0.4 V is ascribed to the formation of SEI [27]. The SiO-PPy-500 has outstanding first discharge/charge capacity at 2058.6/1280 mAh/g (Figure 3a). Compared with an ICE of 54.8% for pure SiO (Figure S4), an enhanced ICE of 62.2% was recorded for SiO-PPy-500. The irreversible capacity loss is attributed to the formation of the SEI passivation film due to the irreversible reaction with electrolyte and the formation of Li_2_O and Li_4_SiO_4_ [28,29]. This might be solved by pre-lithiating the active materials. Figure 3b shows the rate performance of pure SiO and SiO-PPy-500 electrodes cycled ranging from 0.1 to 2 C. The SiO-PPy-500 delivered a capacity of 1127.5 mAh/g at the rate of 0.1 C, and then gradually decreased to 649.9 mAh/g at the rate of 0.5 C, and 487.0 mAh/g at the rate of 1 C. Even at a high rate of 2 C, a reversible capacity of 317.6 mAh/g was still achieved. Notably, when the rate was restored to 0.1 C, the SiO-PPy-500 composite electrode showed a capacity of 1145.7 mAh/g, completely recovering its initial capacity. The results are remarkably superior to that of pure SiO, with a continuous capacity decrease at each current density in the same range. The results show that the SiO-PPy-500 displays an excellent rate performance with a stable cycling behavior at different current densities. Figure 3c and Figure S5 show the cycling stability of the SiO-PPy-500 composite electrodes and pure SiO electrode. After the first cycle at a rate of 0.05 C for the formation step, the cells were then cycled at 0.1 C over 50 cycles between 0.01 and 1.0 V. The SiO-PPy-500 electrodes had much higher reversible specific capacities of 1090.2 mAh/g after 50 cycles than that of the pure electrodes, which was 771.6 mAh/g for the pure SiO electrode. The corresponding capacity retention was 83.5% vs. 66.9% based on the second cycle. 

To investigate the mechanism for improving the electrochemical performance, electrochemical impedance spectra (EIS) measurement was completed on pure SiO- and SiO-PPy-500-based electrodes after discharge/charge for 50 cycles. As shown in Figure 3d, the charge transfer resistance of the SiO-PPy-500 electrode was significantly smaller than that of the SiO electrode, confirming that SiO-PPy-500 has better diffusion capability of Li^+^ and higher electro-conductivity. Long-term cycling tests clearly showed the effect of the high graphitization carbon coatings on the cycle performance of the SiO-PPy-500 composite at a higher current rate (0.3 C) (Figure 3e). A higher specific capacity of 514.6 mAh/g after 500 discharge/charge cycles was achieved, which was much higher than that of traditional graphite anode materials. In addition, the SiO-PPy-500 demonstrated a rapid increase in stabilized efficiency greater than 99.5% after only five cycles, including the first formation cycle, and was very stable in the remaining cycles. This cycling efficiency result is quite promising for the assembly of alloy anodes into full cells. 

We also changed the mass ratio of SiO and PPy to 8:2 to study the effect on the performance of the resulting composite electrodes. The results are shown in Figures S6 and S7. The specific capacity of this electrode was 1069.7 mAh/g after 50 cycles at 0.1 C, and 744.6 mAh/g after 200 discharge/charge cycles at 0.3 C. When SiO-PPy-500 (7:3) was used, the gravimetric capacity of SiO was lower due to the lower carbon content. The higher the carbon content in a pyrolyzed sample, the higher the electronic conductivity, and the better the resistance to volume expansion during the discharge/charge process. 

In addition, sucrose, as conventional carbon coating material, was used and compared in this work (SiO–C–500). The cycling performance of this electrode is shown in Figure S8a. We found that the capacity of the conventional SiO–C–500 electrode was significantly lower when sucrose was used, at only 455.4 mAh/g after 50 cycles at 0.1 C. The charge transfer resistance of the SiO–C–500 electrode was significantly higher than that of the SiO–PPy–500 electrode (Figure S8b), demonstrating a higher charge transfer resistance of the interface and lower electric conductivity. This is because sucrose molecules do not have π–π stacking structures; therefore, they cannot obtain high graphitization at a low sintering temperature. The amorphous carbon provides neither significantly improved conductivity of the SiO materials, nor surface protection for the SiO materials. (Figure S9).

**Figure 4:**
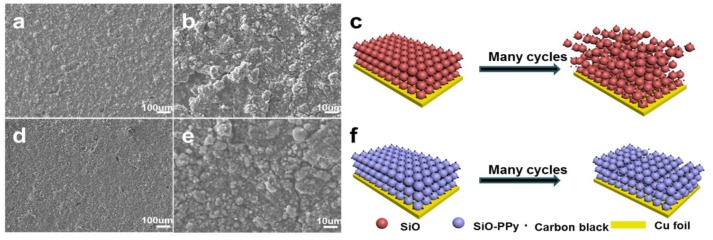
(**a**–**c**) Scanning electron microscopy (SEM) images and corresponding schematic illustration of the pure SiO electrode after 50 cycles at a current density of 0.1 C; (**d**–**f**) SEM images and corresponding schematic illustration of SiO–PPy–500 electrode after 50 cycles at a current density of 0.1 C.

To further analyse the graphite carbon coating effect on the whole electrode during cycling, the cell was disassembled after 50 cycles at 0.1 C and characterized by SEM, as shown in Figure 4. The SEM image after 50 cycles shows the structural degradation of pure SiO (Figure 4a,b). The electrode surface shows obvious cracking, and the laminate lost contact with the current collector. In contrast, from Figure 4d,e, the SiO–PPy–500 electrode features a flat and compact surface in a large domain, maintaining tight contact with the current collector under the same testing conditions. The highly improved cycling performance of the SiO–PPy–500 composite can be attributed to the high graphitization carbon coatings, which improve electron transport, and support fast Li diffusivity. In addition, the coating layer can act as a buffer layer to minimize the volume expansion of the SiO without rupture, thereby avoiding particle pulverization and loss of contact with carbon black or the current collector. By sharp contrast, no sub-micrometer particles were observed in the pure SiO electrode, indicating the serious pulverization of the SiO component. Without graphite carbon coatings, SiO particles cracked under stress of the dimensional changes, leading to degraded cycling performance.

**Figure 5:**
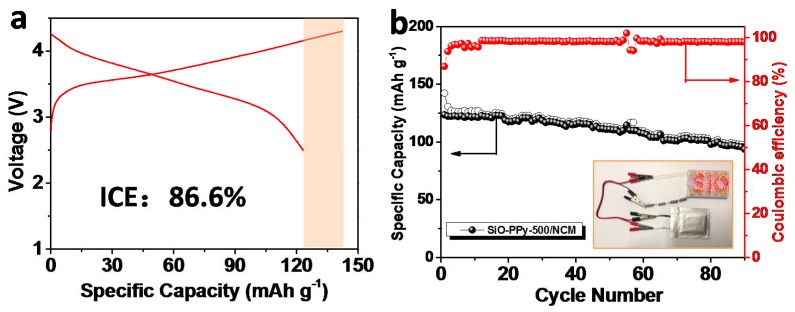
(**a**) Galvanostatic discharge/charge profiles of the prelithiated SiO-PPy-500|NCM full-cell in the first cycles at 1 C and (**b**) cycling performance for 90 cycles at 1 C. The inset shows an array of commercial red light-emitting diodes (LEDs) for SiO powered by the assembled full-cell.

To evaluate the validity of the graphitization core-shell SiO–PPy–500 composite, upon integration with Li[Ni_0.5_Co_0.2_Mn_0.3_]O_2_ (NCM) (half-cell data in Figure S10), full-cells were tested in the voltage range of 2.5 to 4.3 V at a current density of 1 C for 90 cycles (1 C = 160 mA/g) (Figure 5). Before assembling the full-cell, we prelithiated the anode electrode by introducing lithium ions into the SiO materials using electrical shorting with lithium metal foil in the electrolyte [30]. The detailed process is outlined in the experiment section. For the first cycle, the charge and discharge capacities were 142.2 and 123.5 mAh/g, respectively, corresponding to an ICE of 86.6%. After 90 cycles, the full cell delivered a discharge capacity of 93.7 mAh/g, and the capacity retention was 75.9%. We also calculated that the gravimetric energy density of this SiO–PPy–500/NCM full-cell, considering the weight of the anode and cathode materials. From ED = {(*C*_cathode_ × *C*_anode_)/(*C*_cathode_ + *C*_anode_)}*V*_nominal_ [30], the capacity (*C*_cathode_ = 160 mAh/g and *C*_anode_ = 1090 mAh/g) and the voltage (3.6 V), our full-cell had an energy density of 502 Wh/kg, which is much higher than the traditional graphite-based cell. This SiO–PPy–500-based electrode showed good electrochemical performance in the full-cell. 

## 3. Conclusions

In summary, a π–π stacked PPy polymer precursor was used as a coating for SiO materials. The pyrolysis of polymer coating at low to medium temperatures led to a highly graphitized coating on the SiO surface. The electrode based on SiO–PPy–500 material achieved excellent cycling stability. The pyrolyzed PPy coating on the surface of SiO formed a highly graphitized carbon layer that provided a conducting network on SiO materials and a robust layer to prevent electrolyte reaction with the SiO surface. The carbon layer maintained the conductivity of the electrode and acted as an excellent matrix to accommodate the volume expansion during the discharge/charge cycles. The π–π stacked polymer precursor coating is a promising method to modify the surface of Si-based materials for high-capacity LIB anode applications.

## Supplementary Materials

The following are available online at http://www.mdpi.com/2073-4360/10/6/610/s1, Experiment details; SEM images of pure SiO; TGA analysis of SiO-PPy-500; discharge/charge curves of pure SiO at 0.1 C; cycling performance of NCM; TGA analysis and cycling performance of SiO-PPy-500 (SiO to PPy ratio is 8:2); Cycling performance of SiO-C-500; TEM characterization; and TGA analysis.
