# Supplementary material for: Highly Graphitized Carbon Coating on SiO with a π–π Stacking Precursor Polymer for High Performance Lithium-Ion Batteries
Source: Polymers (Basel). 2018 Jun 4;10(6):610. doi: 10.3390/polym10060610 (PMC6403587; doi:10.3390/polym10060610)
Supplement: Supplementary file 1 [file polymers-10-00610-s001.pdf]

Supporting Information

Highly Graphitized Carbon Coating on SiO with a π-π Stacking Precursor Polymer for High Performance Lithium-ion Batteries

Shan Fang, [1,2] *Ning Li,*[1] *Tianyue Zheng,* [1] *Yanbao Fu,* [1] *Xiangyun Song,* [1] *Ting Zhang,* [1]
Shaopeng Li,[2] *Bin Wang*[1], *and Xiaogang Zhang**,[2] *Gao Liu,**,[1]

[1]Energy Storage and Distributed Resources Division, Energy Technologies Area, Lawrence Berkeley National Laboratory, Berkeley, California 94720, United States. E-mail:

[2]Jiangsu Key Laboratory of Electrochemical Energy Storage Technologies, College of Material Science and Engineering, Nanjing University of Aeronautics and Astronautics, Nanjing, 210016, P. R. China.

*azhangxg@nuaa.edu.cn (X. Zhang); gliu@lbl.gov (G. Liu)

Experimental

Preparation of the SiO-PPy composite: Silicon monoxide was purchased from Sigma-Aldrich (~325 mesh). The SiO powders were the first ball milled at 400 rmp for 8h to decrease the particle size from ~10 micrometers to submicrometers. Poly (1-pyrenemethyl methacrylate) was synthesized according to our previous literature. First, 90 mg PPy was dissolved in 30 ml tetrahydrofuran (THF) to obtain a transparent solution. Then 210 mg ball milled SiO added into the above solutions and sonication for 30 mins and stirred for 12h. The solution was then dried by rotary evaporation instrument. Following this, the products were sintered at 400-600°C for 5h in argon to obtain the final products. The SiO-PPy-500 is synthesis with the mass ratio of SiO and PPy is 7:3. The SiO-C-500 prepared in the same way by using sucrose instead of PPy polymer and the mass ratio is 7:3.

Electrochemical tests

The slurry was prepared by mixed the samples, carbon black, PAA-Li binder with a mass ratio of 7:2:1, and DI water as a solvent. The slurry was uniformly coated on a copper foil as the working electrode. The mass

loading of the active material was about 1.6~1.9 mg cm^{-2}. The electrolyte solution was purchased from BASF, which was composed of 1 M $LiPF_6$ solution in a mixture of diethyl carbonate (DEC) and ethylene carbonate (EC) (1:1 w/w) containing 30% (V) fluoroethylene carbonate (FEC). Celgard 2400 separator is obtained from Celgard. The performance of the assembled 2325 coin cells was evaluated with Maccor Series 4000 Battery Test system in a thermal chamber at 30 °C. The cut-off voltage of cell testing is between 1.0 V and 0.01V, the theoretical specific capacity was assumed of 1,000 mAh g^{-1} for SiO.

The Li(Ni0.5Co0.2Mn0.3)O2 (NCM) electrode was prepared by mixing NCM, Super P, and polyvinylidene difluoride (PVDF) dissolved in N-methyl-2-pyrrolidone (NMP) in a ratio of 90: 5: 5 to form a slurry, which was then pasted on an Al foil. After drying under vacuum at 110 °C, the electrodes were tested in coin cells with Li metal as the counter and reference electrodes. The mass loading is ~11.5 mg cm^{-2}. The voltage is between 2.5-4.3V, the electrolyte of this half and full cell is the same as SiO composite half-cell. Pre-lithiating SiO-PPy-500 is processed by introducing an electrical shorting with lithium metal foil. In briefly, the SiO composite electrode was direct contact with lithium foil for appropriate time, in this work we use 20 min, the electrolyte is 60 uL, after pre-lithiation, the electrode was used to make a full cell with NCM.

Characterization of materials

Scanning electron microscopy (JSM-7500F) and transmission electron microscopy (NCEM) was used to measure the microstructure of the composite. The surface areas of the powder were characterized by a Brunauer-Emment-Teller (BET) N_2 adsorption method with a Micrometritic tristar surface area and porosity analyzer. Thermogravimetric analysis was performed on a TGA instrument (NETZSCH STA 409 PC) with a heating rate of 10 °C min^{-1} from 30 to 700 °C under ambient conditions. X-ray diffraction (XRD) (Bruker D8 advance) with Cu Ka radiation was used to characterize the crystal structures of the samples. Raman spectrum was performed with 488 nm wavelength to check the compositions and the carbon content of the product were check by EA (Vario EL-III), and Attenuated Total Reflectance Fourier transforms infrared (ATR-FTIR) spectroscopy was used to check the structure of pure PPy after sintering at a different temperature. The X-ray photoelectron spectroscopy (XPS) analysis was carried out through a Perkin–Elmer PHI 550 spectrometer with Al Ka (1486.6 eV) as the X-ray source.

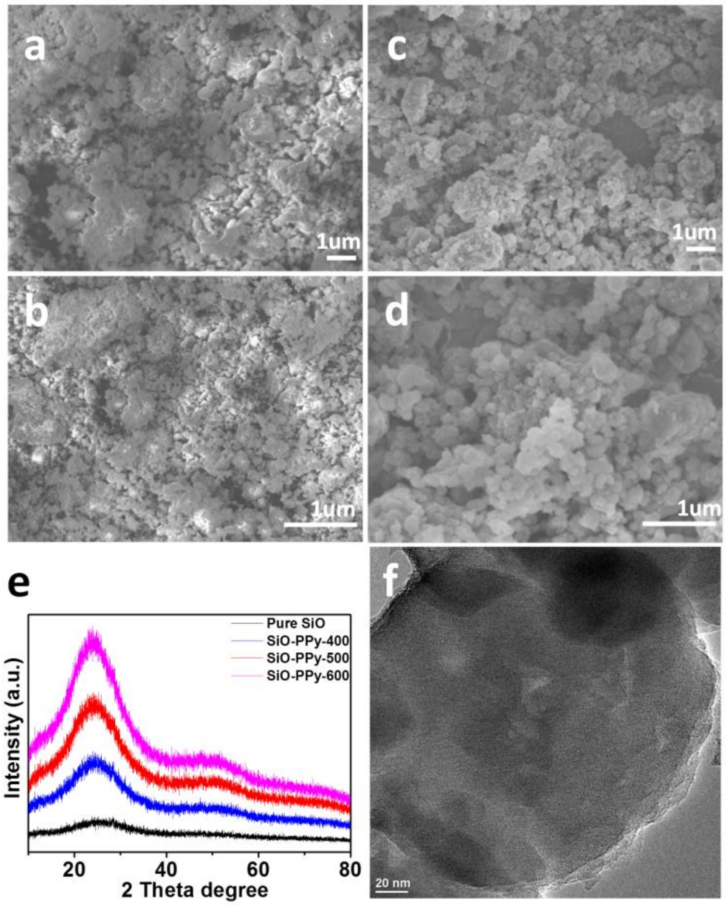

Figure S1. The SEM images of (a, b) pure SiO; (c, d) SiO-PPy-500 (7:3); (e) The XRD spectra of pure SiO and SiO-PPy treated at 400℃, 500℃ and 600℃ ; (f) The TEM image of SiO-PPy-500.

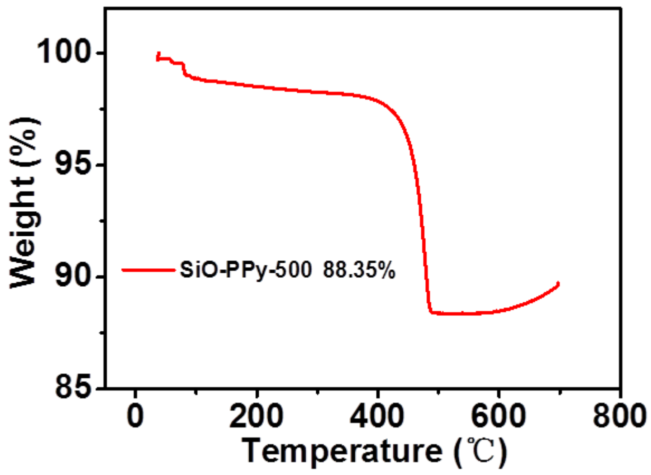

Figure S2. TGA curve of SiO-PPy-500 composite sample measured from 30 to 700°C in flowing air.

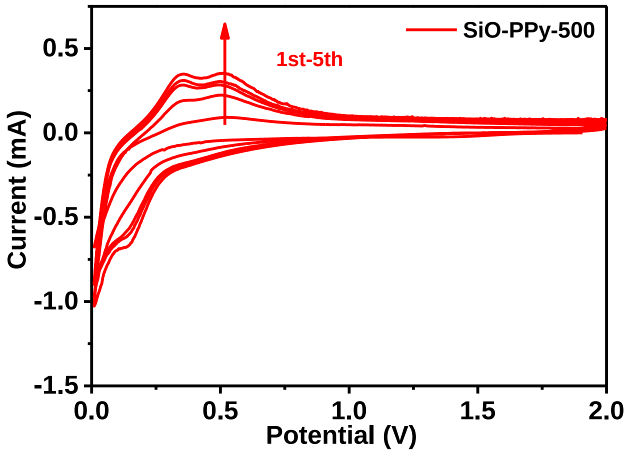

Figure S3. Cyclic voltammetry of SiO-PPy-500 between 2.0~0.01V with a scan rate of 0.1 mV s^{-1}.

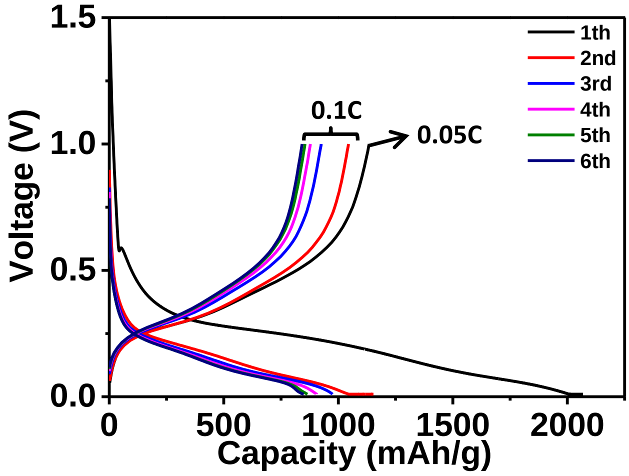

Figure S4. Discharge/charge profiles of Pure SiO measured at 0.05C for the first cycles and then 0.1C for other cycles.

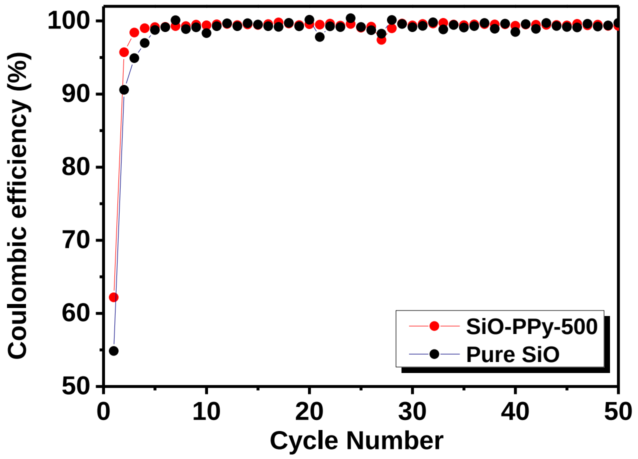

Figure S5. Coulombic efficiency of Pure SiO and SiO-PPy-500 after 50 cycles at 0.1C.

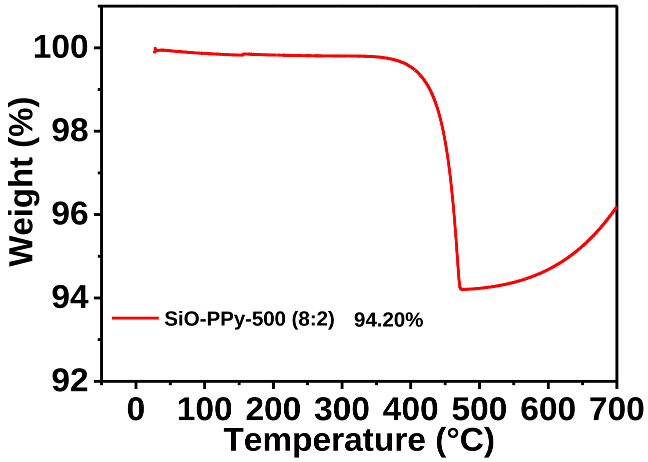

Figure S6. TGA curve of SiO-PPy-500 composite sample (the mass ratio of SiO to PPy is 8:2) measured from 30 to 700°C in flowing air.

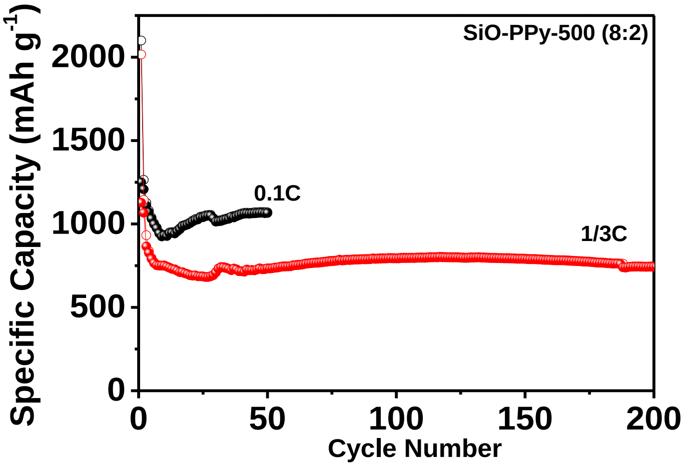

Figure S7. Cycling performance of SiO-PPy-500 (mass ratio is 8:2) at current densities of 0.1C and 1/3 C. The specific capacity of this electrode is 1069.7 after 50 cycles at 0.1C and 744.6 mAh g^{-1} after 200 discharge/charge cycles at 1/3 C, respectively.

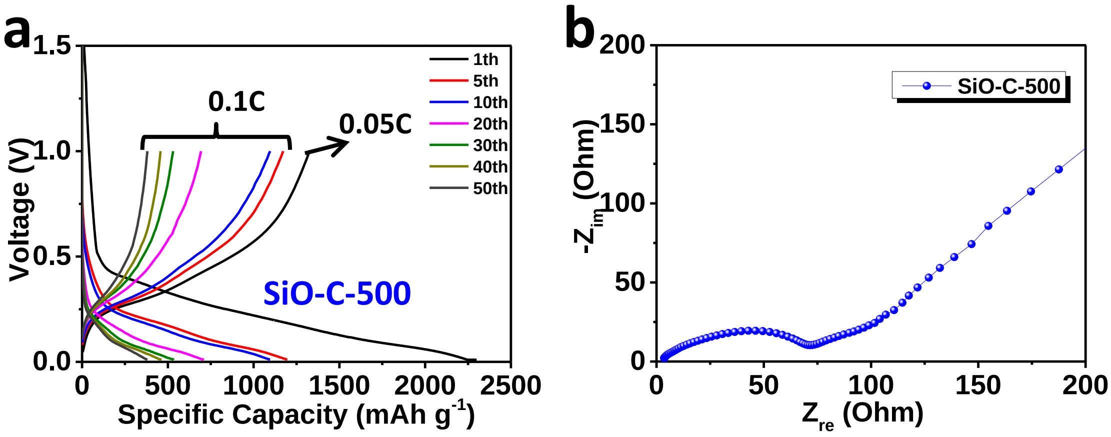

Figure S8. (a) Discharge/Charge curves of SiO-C-500 at different cycles. (b) Nyquist plots of SiO-C-500 electrode after 50 cycles.

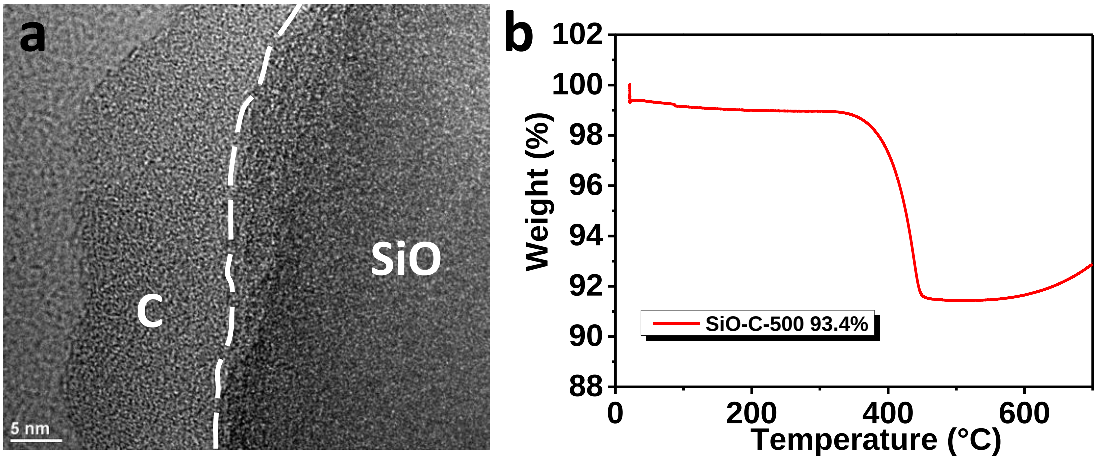

Figure S9. (a) HRTEM of SiO-C-500 and (b) TGA curves.

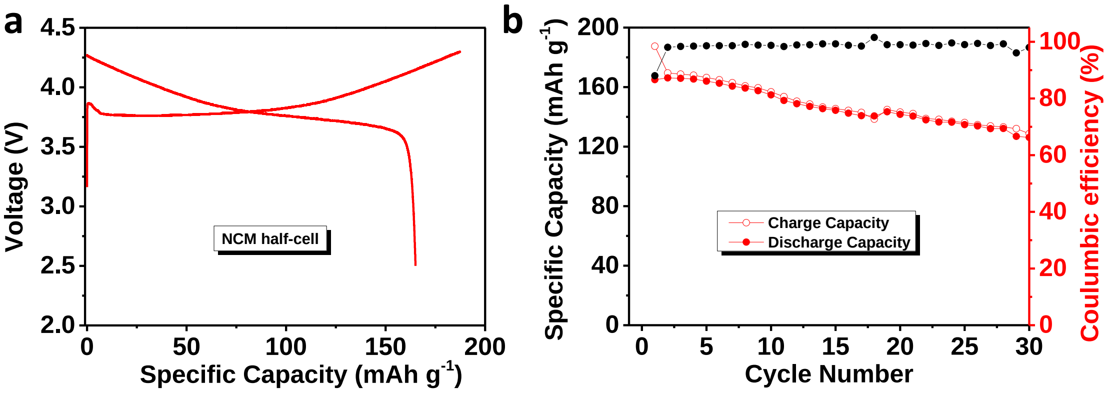

Figure S10. (a) Charge/Discharge curves of NCM (523) measrued at 0.1C (1C=160 mA g^{-1}). (b) Cycling performance of NCM at 0.1C for 30 cycles at voltage range of 2.5-4.3V.
